# Ultrafast multidimensional Laplace NMR for a rapid and sensitive chemical analysis

**DOI:** 10.1038/ncomms9363

**Published:** 2015-09-18

**Authors:** Susanna Ahola, Vladimir V Zhivonitko, Otto Mankinen, Guannan Zhang, Anu M. Kantola, Hsueh-Ying Chen, Christian Hilty, Igor V. Koptyug, Ville-Veikko Telkki

**Affiliations:** 1 Faculty of Science, NMR Research Group, University of Oulu, PO Box 3000, 90014 Oulu, Finland; 2 Laboratory of Magnetic Resonance Microimaging, International Tomography Center SB RAS, Instututskaya Street 3A, 630090 Novosibirsk, Russia; 3 Department of Natural Sciences, Novosibirsk State University, Pirogova Street 2, 630090 Novosibirsk, Russia; 4 Department of Chemistry, Texas A&M University, 3255 TAMU, College Station, Texas 77843-3255, USA

## Abstract

Traditional nuclear magnetic resonance (NMR) spectroscopy relies on the versatile chemical information conveyed by spectra. To complement conventional NMR, Laplace NMR explores diffusion and relaxation phenomena to reveal details on molecular motions. Under a broad concept of ultrafast multidimensional Laplace NMR, here we introduce an ultrafast diffusion-relaxation correlation experiment enhancing the resolution and information content of corresponding 1D experiments as well as reducing the experiment time by one to two orders of magnitude or more as compared with its conventional 2D counterpart. We demonstrate that the method allows one to distinguish identical molecules in different physical environments and provides chemical resolution missing in NMR spectra. Although the sensitivity of the new method is reduced due to spatial encoding, the single-scan approach enables one to use hyperpolarized substances to boost the sensitivity by several orders of magnitude, significantly enhancing the overall sensitivity of multidimensional Laplace NMR.

## Abstract (web summary)

Laplace NMR provides complementary information to traditional NMR, such as details of molecular motion. Here, the authors report a correlation experiment capable of providing information on the physical environment of molecules while enhancing the chemical resolution and greatly reducing the experiment times.

## Graphical abstract

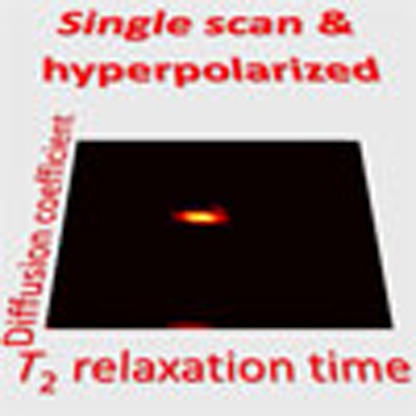

Nuclear magnetic resonance (NMR) spectroscopy is one of the most powerful analytical techniques in chemical sciences^1^. NMR is also one of the very few methods for measuring molecular self-diffusion without an invasive tracer^2^, and the relaxation experiments reveal the effect of random molecular motion on the recovery of initially perturbed nuclear magnetization^3^. Although the frequency content of conventional, oscillating NMR signal is analysed by a Fourier transform, the relaxation and diffusion data consist of exponentially decaying components, and the distribution of diffusion coefficients or relaxation times can be extracted from the experimental data by an inverse Laplace transform^2^. Consequently, these methods can be referred to as Laplace NMR (LNMR).

Like in traditional NMR spectroscopy, the resolution and information content of LNMR can be increased by a multidimensional approach^2^,^4^,^5^. The approach has entered routine use only in recent years, after the development of a sufficiently reliable and robust multidimensional Laplace inversion algorithm^6^,^7^,^8^. However, the conventional scheme of multidimensional NMR based on the repetition of the experiment with varying evolution delay leads to a long experiment time, restricting the investigation of fast processes. The need for multiple repetitions also practically prevents the utilization of significant sensitivity gain provided by nuclear spin hyperpolarization methods, such as dynamic nuclear polarization (DNP)^9^, parahydrogen-induced polarization (PHIP)^10^ and spin-exchange optical pumping^11^.

As a solution to these problems, we introduce a concept of ultrafast multidimensional LNMR, which is based on continuous spatial encoding. Recently, these principles have been successfully applied in ultrafast multidimensional NMR spectroscopy^12^,^13^,^14^ (also with hyperpolarization^15^), in one-dimensional (1D) LNMR experiments^16^,^17^, as well as for correlating diffusion and spectroscopic data^18^. Here, we describe an ultrafast diffusion–*T*_2_ relaxation (*D*–*T*_2_) correlation experiment. Supported by our recent work^19^, in which we proposed an ultrafast *T*_1_–*T*_2_ relaxation correlation experiment, it proves that the principles are applicable to a broad range of multidimensional LNMR experiments and can be used to efficiently correlate relaxation times and diffusion coefficients as well as to investigate chemical exchange phenomena.

## Results

### Ultrafast *D*–*T*_2_ correlation LNMR experiment

**Figure 1:**
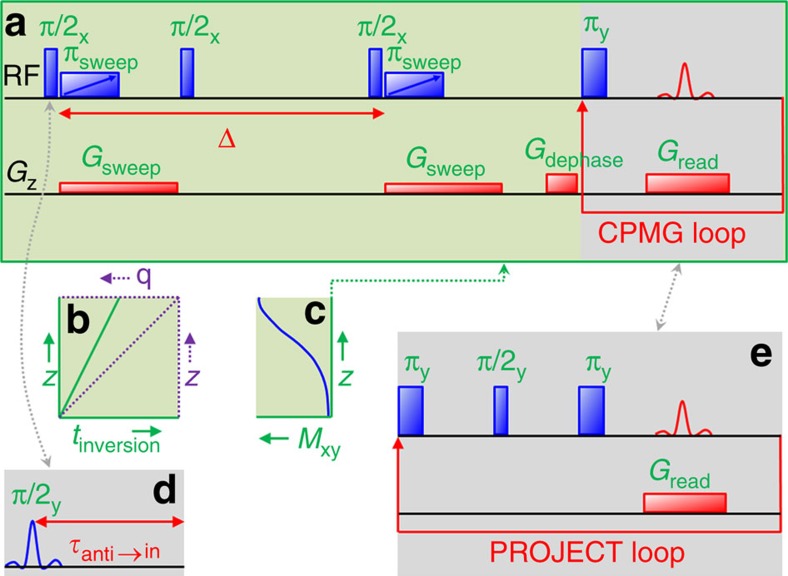
Ultrafast *D*–*T*_2_ correlation LNMR experiment. (**a**) Pulse sequence. (**b**) Spatial dependence of the inversion time *t*_inversion_ and the value of wave vector *q* due to the frequency-swept radio frequency and gradient pulse pair. (**c**) Transverse magnetization profile after the diffusion encoding. (**d**) A soft pulse and a delay replacing the first π/2 pulse to convert the antiphase signal to in-phase in PHIP experiments. (**e**) PROJECT loop replacing the CPMG loop in order to eliminate J modulation.

The ultrafast *D*–*T*_2_ correlation experiment (Fig. 1a) begins with spatial encoding of diffusion data along the longitudinal (*z*) axis of a sample tube, similar to single-scan diffusion-ordered spectroscopy^17^. After the first π/2 pulse, spins at different *z* positions experience the frequency-swept refocusing π-pulse at different times because of the simultaneously applied magnetic field gradient pulse. Consequently, the value of the wave vector *q* (proportional to the strength of the gradient)^2^ becomes linearly dependent on the position, being zero at the top and maximum at the bottom (Fig. 1b). Subsequently, the magnetization is stored along the longitudinal direction for the mixing (diffusion) period, followed by the second pair of the radio frequency (RF) frequency-swept and gradient pulses. Because of diffusion, the resulting stimulated echo is the most intense at the top and weakest at the bottom (Fig. 1c). The final *T*_2_-encoding part comprises a Carr-Purcell-Meiboom-Gill (CPMG) loop^20^, and the magnetization profile along the *z* direction is imaged at each CPMG echo point similar to multiple-echo magnetic resonance imaging (MRI)^21^. After Fourier transform in the spatial frequency (*k*) dimension, the resulting data set is analogous to that obtained from the conventional *D*–*T*_2_ correlation experiment^22^ comprising pulsed-field-gradient stimulated-echo^23^ and CPMG blocks. The ultrafast experiment, however, is measured in a single scan. The number of points in the indirect (diffusion) dimension typically varies from tens to hundreds, which are collected in a repetitive manner in the conventional experiments. Therefore, the experiment time in the ultrafast version can be one to two orders of magnitude (or more) shorter. The price to pay is the reduced sensitivity because of the spatial encoding^14^. However, if the concentration of the sample is high, the sensitivity is not an issue in high-field NMR spectrometers, and the sensitivity losses can be alleviated by a moderate amount of averaging or overcompensated through the use of hyperpolarized substances, as we show below. Spatial encoding also requires a homogeneous sample, although slight inhomogeneity can be compensated in post-processing (see below).

### Resolving different physical environments of molecules

**Figure 2:**
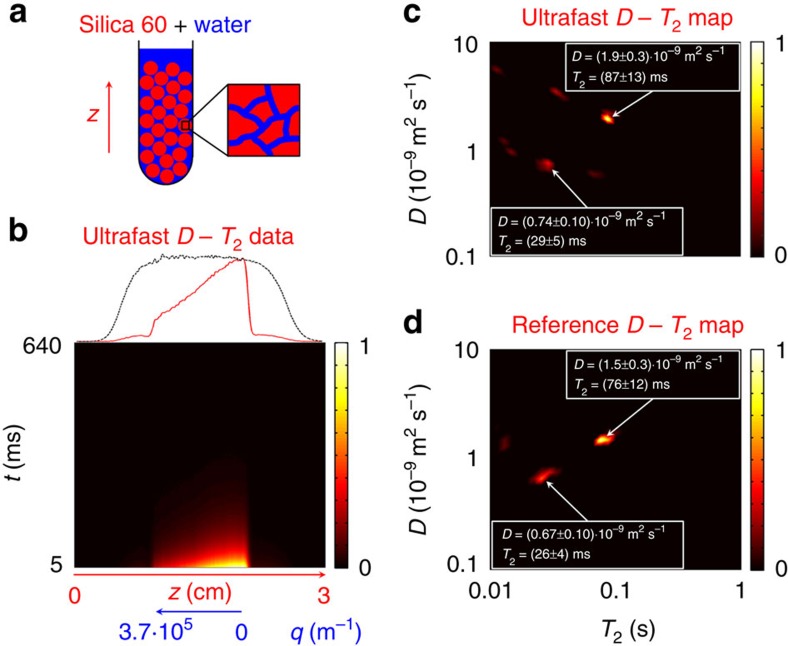
Resolving different physical environments of molecules. (**a**) Schematics of the sample consisting of porous silica gel 60 powder immersed in water (1% H_2_O in D_2_O). (**b**) Experimental ultrafast *D–T*_2_ data after the Fourier transform in the spatial frequency dimension. The first row (red) is shown on the top along with the coil sensitivity profile (black). (**c**) Ultrafast *D–T*_2_ map including one peak arising from water in the pores (*D*=0.74·10^−9^ m^2^ s^−1^, *T*_2_=29 ms) and the other from water between the particles (*D*=1.9·10^−9^ m^2^ s^−1^, *T*_2_=87 ms). The map is the result of a 2D Laplace inversion of experimental data corrected using the coil sensitivity profile in the region affected by the frequency-swept pulse (*z*=0.96–1.99 cm). (**d**) Corresponding reference map obtained in the conventional *D–T*_2_ correlation experiment. The experiments were carried out at 300 MHz ^1^H frequency. The total time in the conventional experiment was 46 min, using eight scans per increment, while only 2 min 30 s were required with a total of 32 scans in the ultrafast experiment.

In the first experimental demonstration, we show that, contrary to a ^1^H NMR spectrum, the ultrafast *D*–*T*_2_ correlation experiment resolves differing physical environments of water molecules in a sample consisting of water and silica gel 60 porous powder with an average pore diameter of 6 nm and particle size of 60–200 μm (Fig. 2a). The experimental data after Fourier transform in the spatial frequency dimension are shown in Fig. 2b. The observed diffusion-encoded magnetization profile along the *z* direction is weighted by the excitation-detection profile of the coil and the slight sample heterogeneity. To eliminate this weighting, we measured in a separate experiment the coil excitation-detection profile, that is, the 1D MRI of the sample along the *z* axis (Fig. 2b), with the same imaging parameters as in the CPMG loop of the ultrafast *D*–*T*_2_ experiment. Each row in the *D*–*T*_2_ data set was then divided along the *z* direction by this profile. Before the two-dimensional (2D) Laplace inversion, the data outside the region affected by the frequency-swept inversion pulse were also removed, and the *z* axis was converted into a *q* axis using the linear relationship between these two quantities. The resulting *D*–*T*_2_ map includes two dominant peaks: one, with smaller *D* and shorter *T*_2_, arising from water in the pores, and the other from the bulk water in the spaces between the particles of the porous material. There are also some additional minor peaks that arise from imperfectly compensated sample heterogeneity and noise. The largest artefact has an amplitude of about 28% of the highest peak. The ultrafast experiment is more sensitive to local field inhomogeneities along the sample axis than the conventional one, because the various evolution delays are encoded in the layers of the sample, whereas in the conventional experiment the signal corresponding to a single evolution time is measured from the entire sample volume inside the NMR coil. Artefacts due to background gradients in a heterogeneous sample could be removed by using a bipolar gradient^24^ in the ultrafast *D*–*T*_2_ experiment. In this alternative implementation, the *G*_sweep_ gradient would be replaced with a pair of gradients with opposite amplitudes. The π_sweep_ pulses associated with the gradients would have the same sweep direction. We also carried out a conventional *D*–*T*_2_ correlation experiment to serve as a reference. The resulting *D*–*T*_2_ map (Fig. 2d) shows the same dominant peaks as the ultrafast map and, within the error limits, results in the same *D* and *T*_2_ values, proving that the ultrafast method works. It is notable that, despite the fourfold number of scans, the measurement time for the ultrafast experiment was 18 times shorter than for the conventional experiment.

### Improved chemical resolution

In the following, we will demonstrate that chemical resolution lacking in the NMR spectrum of hydrocarbons can be revealed by the ultrafast *D*–*T*_2_ experiment. However, homonuclear scalar coupling present in these molecules modulates the CPMG echo amplitudes and severely complicates the *T*_2_ data. This problem can be solved by replacing the CPMG block with a PROJECT (Periodic Refocusing of J Evolution by Coherence Transfer)^25^ block (Fig. 1e). The PROJECT is a cyclic analogue of the perfect echo experiment^26^, in which a π/2 pulse at the midpoint of a double spin echo refocuses the J modulation.

**Figure 3:**
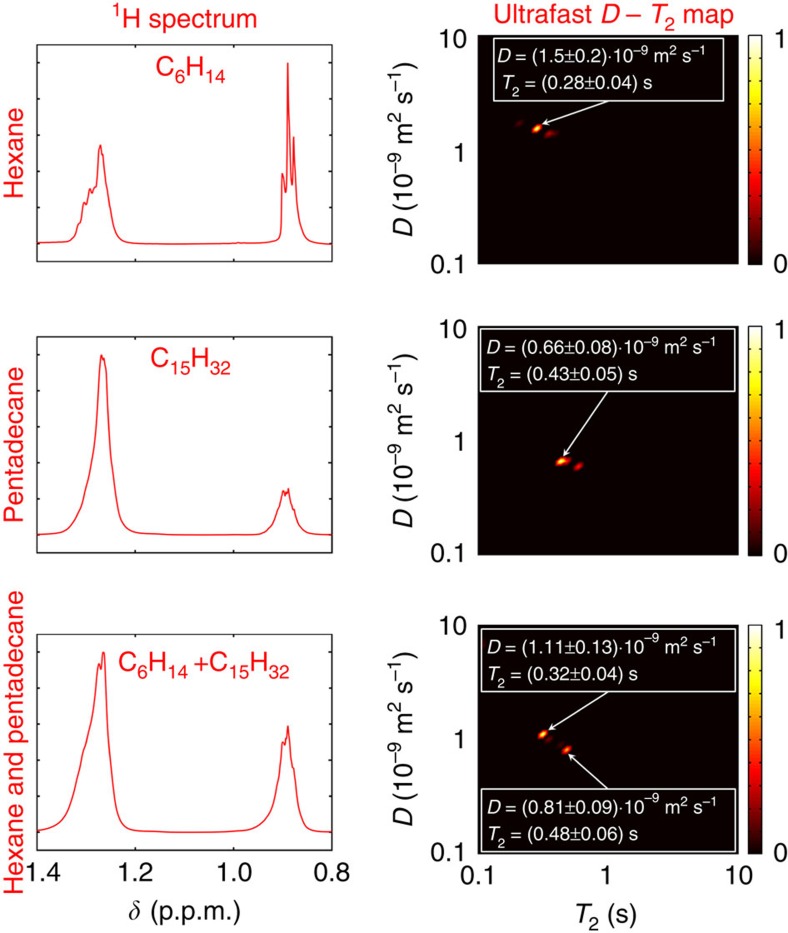
Improved chemical resolution. ^1^H NMR spectra and ultrafast (PROJECT-based) *D*–*T*_2_ maps of 1.65 M hexane, 0.79 M pentadecane and a mixture of 1.36 M hexane and 0.65 M pentadecane in CCl_4_. Although the compounds are not resolved in the spectrum of the mixture, they are resolved in the *D*–*T*_2_ map. The experiments were carried out on a 600-MHz NMR spectrometer.

The ^1^H NMR spectrum of hexane includes two peaks, one arising from hydrogens in methyl (CH_3_-) groups at 0.9 p.p.m. and the other from methylene (-CH_2_-) groups at 1.3 p.p.m., and the same groups resonate at the same frequencies also in the case of pentadecane. Consequently, these two chemicals are not resolved in the ^1^H spectrum of their mixture (Fig. 3). However, the ultrafast *D*–*T*_2_ experiments (experimental data in Supplementary Fig. 1) result in maps that are unique for each compound, with larger *D* and shorter *T*_2_ for hexane than for pentadecane, and the compounds are resolved also in the map of the mixture (Fig. 3). The amplitudes of the two signals are roughly equal, as expected based on the concentrations used. Importantly, the *D* values obtained in the ultrafast measurements performed on individual solutions are in good agreement with the values measured by the standard pulsed-field-gradient stimulated-echo NMR (hexane: 1.62·10^−9^ m^2^ s^−1^, pentadecane: 0.66·10^−9^ m^2^ s^−1^), confirming the reliability of the ultrafast experiments. At the same time, for the mixed hexane-pentadecane sample, the observed *D* value of hexane in the mixture, (1.11±0.13)·10^−9^ m^2^ s^−1^, is smaller than that of hexane in the reference individual sample, which is physically reasonable according to the scaling laws for diffusion coefficients in mixtures of alkanes (large pentadecane molecules hinder the diffusion of hexane)^27^. As for *T*_2_, the imaging magnetic field gradients in the PROJECT block of the ultrafast experiments make the observed values significantly shorter than in the standard PROJECT experiment without a gradient because of two factors well-known from, for example, *T*_2_ maps measured with MRI or in *T*_2_ measurements performed using single-sided NMR with an inhomogeneous magnetic field: diffusion in inhomogeneous field speeds up the echo attenuation^2^ and the gradients make the experiment more sensitive to *B*_1_ inhomogeneity^28^. Although in standard PROJECT experiments of the hexane and pentadecane samples the observed *T*_2_ value of hexane (3.0 s) was higher than that of pentadecane (1.2 s), consistently with the scaling law for relaxation times^29^, in the ultrafast experiment it is the other way around because of above-mentioned reasons. We note that the *T*_2_ shortening is minor in the water/silica sample (Fig. 2) because strong local field inhomogeneities caused by the porous material are present both in the ultrafast and reference experiments.

It is also worth to note that the two compounds are not resolved in the 1D *T*_2_ and *D* distributions (Supplementary Fig. 1e and f) obtained by the Laplace inversion of the first row or column of the 2D ultrafast data, indicating that the 2D approach increased the resolution of the experiment.

### Boosting sensitivity by hyperpolarization

In principle, the sensitivity of LNMR can be increased by several orders of magnitude by means of nuclear spin hyperpolarization^9^,^10^,^11^, broadening the applicability of the method to low concentration samples. However, the conventional multidimensional LNMR approach practically prevents the use of hyperpolarized substances, because the experiment has to be repeated multiple times with varying evolution time. Hyperpolarization should be regenerated before each repetition, which is extremely laborious and time consuming, and it may even take hours in the case of some implementations of DNP^9^. Furthermore, the polarization level might vary among repetitions, causing serious artefacts in the experimental data. The ultrafast multidimensional approach, realized in a single-scan manner, can overcome these problems.

**Figure 4:**
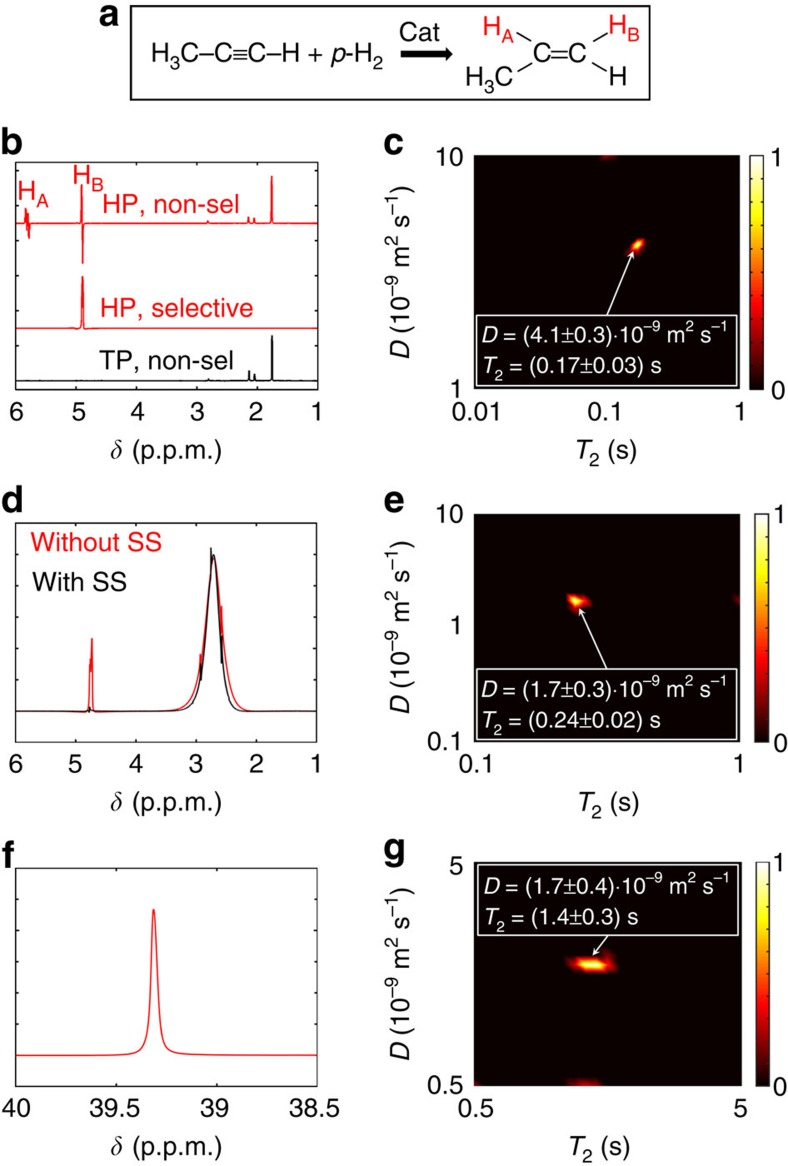
Boosting sensitivity by hyperpolarization. (**a**) Hydrogenation of propyne into propene with parahydrogen (*p*-H_2_) to produce PHIP. Red symbols indicate the hyperpolarized hydrogens. (**b**) Top: Hyperpolarized (HP) ^1^H NMR spectrum measured right after bubbling a mixture of *p*-H_2_ and propyne through the solution of [Rh(COD)(DPPB)]BF_4_ catalyst in deuterated acetone. The antiphase propene multiplets at 4.9 and 5.8 p.p.m. indicate a strong PHIP effect. Middle: the corresponding spectrum recorded after a selective excitation of the methylene (4.9 p.p.m.) signal followed by a delay converting the antiphase signal into an in-phase signal. Bottom: The spectrum measured after the decay of PHIP hyperpolarization due to relaxation. (**c**) Single-scan *D*–*T*_2_ map of hyperpolarized propene using the selective excitation of the 4.9 p.p.m. signal. The experiment time was only 0.5 s. The PHIP experiments were carried out at 600 MHz ^1^H frequency. (**d**) ^1^H NMR spectra of DNP hyperpolarized DMSO in H_2_O, both with and without solvent suppression (SS). (**e**) Single-scan *D*–*T*_2_ map of hyperpolarized DMSO measured after solvent suppression. (**f**) ^13^C NMR spectrum of DNP hyperpolarized DMSO in H_2_O. (**g**) Corresponding single-scan *D*–*T*_2_ map. The DNP NMR measurements were carried out at 400 MHz for ^1^H and 100 MHz for ^13^C.

First, we produced hyperpolarization using the PHIP method by bubbling a mixture of propyne and parahydrogen (prepared by cooling H_2_ to 77 K in the presence of a paramagnetic material^30^, see the Supplementary Methods) through the solution of hydrogenation catalyst in deuterated acetone. The resulting hydrogenation reaction produced hyperpolarized propene (Fig. 4a). Based on the hyperpolarized and thermally polarized ^1^H spectra shown in Fig. 4b, the sensitivity enhancement factor given by PHIP was estimated to be about 500. The ultrafast *D*–*T*_2_ experiment was modified by replacing the hard π/2 excitation pulse with a selective excitation of the methylene resonance of propene, followed by a delay for converting the antiphase PHIP signal into an in-phase signal (Fig. 1d). This was done to ensure that the opposite components of the antiphase multiplet would not cancel each other when reading the magnetization profile in the PROJECT loop. The single-scan *D*–*T*_2_ map of hyperpolarized propene is shown in Fig. 4c (experimental data in Supplementary Fig. 2). The map expectedly contains a single peak with a realistic *D* value, although the concentration of propene was only about 40 mM, and the experiment time was notably reduced to 0.5 s. In this experiment, the observed *T*_2_ (0.17 s) was exceptionally much shortened as compared with the thermally polarized reference value (9.5 s) because of the effect of very strong read gradients used in the experiment and the large diffusion coefficient of propene. The value would approach the reference value by decreasing the read gradient amplitude.

In the second demonstration, the ultrafast *D*–*T*_2_ measurement was applied to a hyperpolarized spin system prepared via dissolution DNP^9^. A 5-μl sample of dimethyl sulfoxide (DMSO) in D_2_O (v/v 18:7) with 15 mM of 4-hydroxy-2,2,6,6-tetramethylpiperidine 1-oxyl radicals was first hyperpolarized by microwave irradiation of 94.005 GHz at 1.4 K in a field of 3.35 T for about 30 min. Subsequently, the sample was dissolved with superheated water. This sample solution was rapidly transferred to an injection loop, and driven into a flow cell in a 400-MHz NMR magnet using water from a high-pressure pump^31^. ^1^H NMR spectra of hyperpolarized DMSO in H_2_O, both with and without solvent suppression, are shown for reference in Fig. 4d. The final concentration of DMSO was determined to be 34 mM. The broadening of the DMSO signal in the spectra is due to radiation damping^32^, which arises because of the strong hyperpolarized signal. Figure 4e shows the *D*–*T*_2_ map of DMSO, measured in a single scan 3 s after stopping the in- and out-flow of the sample by switching an injection valve. These data were acquired with solvent suppression. It is important to assure that the sample convection after the transport does not disturb the spatial encoding. In these experiments the sample convection was significantly reduced because of the use of flow cell with liquid-driven injection^31^, and single-scan *D*–*T*_2_ test experiments showed that the convection was insignificant after the 3 s stabilization delay. In the homogeneous sample, the observed *T*_2_ value is shortened (*T*_2_ in the thermally polarized reference experiment was 3.0 s), because of the effects described above. The *D* value, which is strongly dependent on DMSO concentration^33^, was found to be (1.7±0.3)·10^−9^ m^2^ s^−1^. This value is in agreement with a reference measurement using a conventional stimulated echo pulse sequence on a stationary, non-hyperpolarized sample at *T*=300 K, which yielded *D*=1.3·10^−9^ m^2^ s^−1^. The agreement of the measured diffusion coefficients indicates that the sample solution was very nearly stationary during the measurement, in agreement with our previous pulsed field gradient-based characterization of high-pressure liquid-driven injection into a flow cell^31^. This demonstration shows that the challenges related to fast transport of hyperpolarized substances and their stabilization before an ultrafast LNMR experiment can be overcome.

Figure 4g shows *D*–*T*_2_ map of the corresponding experiment, in which^13^C nuclei, instead of ^1^H, were hyperpolarized and detected in the ultrafast *D*–*T*_2_ experiment. In this experiment, a final concentration of DMSO of 288 mM was used. The signal-to-noise ratio (SNR) in the *D*–*T*_2_ experiment was rather low (about 24) because of small gyromagnetic ratio of ^13^C (one-fourth of that of ^1^H) and low natural abundance of ^13^C isotope (1%), but, still, the 2D Laplace inversion resulted in the expected single component *D*–*T*_2_ map with a *D* value in good agreement with the ultrafast ^1^H and reference experiments. Again, the observed *T*_2_ value (1.4 s) was shorter than in the thermally polarized reference experiment (2.8 s) because of the use of the read gradients. We stress that this kind of multidimensional LNMR experiment with a low-sensitivity heteronucleus is absolutely unattainable with the conventional technique relying on thermal polarization. Here, the ^13^C signal enhancement was estimated to be about 3,200 by comparing the DMSO signal to the signal obtained from a sample of known concentration in a separate DNP experiment. Consequently, the ultrafast method opens unprecedented prospects of applications of multidimensional LNMR, as DNP is the most universal hyperpolarization method applicable to any NMR active nuclei with sufficiently long relaxation time.

## Discussion

When dealing with LNMR, one has to keep in mind that the inverse Laplace transform of noisy data is an ill-posed problem, meaning that there exist an infinite number of relaxation time or diffusion coefficient distributions consistent with the experimental data^5^. The inversion becomes feasible with the use of regulator smoothing the distributions^34^,^35^. However, the inversion algorithms tend to broaden intrinsically narrow peaks, lowering the resolution, but also to split up intrinsically broad peaks into a series of narrow peaks (the so-called uniform-penalty smoothing, however, provides a satisfactory solution to these problems)^35^. Consequently, it is advisable to confirm the reliability of the results by reference experiments (described above) and simulations. We have physical reasons to assume that both the silica/water sample and the mixed hexane–pentadecane sample produce two rather sharp *D* and *T*_2_ peaks: one from water in the small pores and the other from bulk water in between the particles in the former case, and one from each compound in the latter case. We simulated the *D*–*T*_2_ correlation LNMR data for two components with a zero peak width, varying the peak amplitudes and separation as well as the SNR, and performed 1D and 2D Laplace inversions of the data (see Supplementary Figs 3 and 4 as well as Supplementary Discussion). The results confirmed that the resolution in the LNMR distributions can be improved by increasing SNR, as has been shown earlier^35^, and that it is rather high when SNR is high: if SNR is 1,000, peaks of equal amplitude with relaxation times and/or diffusion coefficients differing by a factor of 1.5 can be resolved. If SNR is 10,000, this factor can be as low as 1.25 (Supplementary Fig. 5). These SNR values are achievable with high field NMR instruments, if the sample concentration is high enough or if signal is amplified by hyperpolarization. On the other hand, systematic errors due to hardware imperfections become more significant relative to the noise variation with increasing SNR, and may become a limiting factor of the accuracy of the method. In any case, the simulations affirm that the compounds of the hexane/pentadecane mixture can be reliably separated in the ultrafast *D*–*T*_2_ experiment, because SNR in the experiment was 8,000 (Supplementary Fig. 1).

As already stated, the acceleration of multidimensional LNMR by the ultrafast method based on spatial encoding leads to sensitivity reduction, which depends on numerous experimental parameters. To obtain an estimate, we carried out both ultrafast and conventional *D*–*T*_2_ correlation experiments on a doped water sample using the same number of scans and almost identical *q* and *t* values. The SNR of the ultrafast experiment determined from the echo amplitude data, *E*(*q*,*t*), was about four times lower than in the reference experiment. However, remarkably, the SNR per unit time was slightly better (by the factor of 1.8) in the ultrafast experiment, as 64-fold number of scans were accumulated in the same experiment time. Pathan *et al.*^36^ have reported a similar sensitivity gain per unit time provided by ultrafast 2D NMR spectroscopy. It is worth noting that the single-scan sensitivity loss factor (4) is much smaller than a typical sensitivity gain of several orders of magnitude given by hyperpolarization methods. Consequently, by combining hyperpolarization with ultrafast techniques, one can increase significantly the overall sensitivity of multidimensional LNMR.

An important question related to sensitivity is: what is the limiting concentration in order to obtain reliable results with ultrafast *D*–*T*_2_ LNMR. The lower concentration limit depends on many factors, including the desired *D* and *T*_2_ resolution (Supplementary Fig. 5), the strength of the magnetic field of the NMR spectrometer, the number of observed nuclei in the molecule and the gyromagnetic ratios. The SNR from the hexane–pentadecane mixture was about 8,000, obtained with 128 scans (Supplementary Fig. 1). The overall concentration was about 2 M. As the SNR in the LNMR experiment should be at least 100 in order to resolve two components (Supplementary Fig. 5), we can estimate that under these conditions the minimum concentration for a single-scan experiment is about 0.3 M. The limit can be decreased moderately by accumulation and substantially (by orders of magnitude) by hyperpolarization.

One limitation of the ultrafast *D*–*T*_2_ experiment is that the frequency-swept refocusing π-pulse does not work perfectly immediately after it has been switched on^14^. This limits the measurement of the echo amplitudes corresponding to very small and very large *q* values simultaneously in a single experiment, and consequently impacts the range of the diffusion coefficients that can be detected. Another limitation is that, although it is necessary to measure only a single data point at each echo maximum in the CPMG loop in the conventional method, the ultrafast method requires collecting a sufficient number of points in the presence of the imaging gradient in order to obtain the magnetization profile along the sample axis. This requirement limits the shortest possible echo time in the CPMG loop, and consequently the shortest observable *T*_2_ value. However, the *T*_2_ limit can be decreased by increasing the imaging gradient strength. The experimental results reported in this manuscript show that, regardless of these limitations, the typical moderate range of *D* and *T*_2_ in liquids falls well within the capacity of the experiment.

The additional elements of the ultrafast *D*–*T*_2_ experiment as compared with the conventional method are the frequency-swept π-pulse and the imaging gradients of the CPMG (or PROJECT) loop. Most modern NMR spectrometers are able to generate shaped pulses and typically provide at least the z gradient; hence, the ultrafast *D*–*T*_2_ experiment can be carried out with standard NMR instrumentation. Setting up the experimental parameters can be somewhat more complicated than in the conventional experiments: first, one has to set the length and amplitude of the diffusion gradient (*G*_sweep_) to correspond to the highest desired *q* value, then the bandwidth of the frequency-swept pulse has to be adjusted to match the Larmor frequencies (linearly dependent on the position due to the presence of *G*_sweep_) of the nuclei in the sensitive region of the radio frequency coil and finally the amplitude of the read gradient, the dwell time and the number of collected points has to be set so that the 1D image has a proper field-of-view and resolution. It is also advisable to include a correction for the coil sensitivity profile, as described above. However, it is possible to automate the measurement process so that, after the desired *D* and *T*_2_ ranges are specified, the measurement programme calculates all the pulse programme parameters and applies the sensitivity profile correction to the measured data.

We believe that the results presented above, along with our recent publication introducing the *T*_1_–*T*_2_ correlation experiment^19^, open a broad field of ultrafast multidimensional NMR, because, although not trivial, the basic blocks of the experiments can be used to develop multiple 2D and even three-dimensional ultrafast LNMR experiments for correlating relaxation times and diffusion coefficients as well as for investigating chemical exchange phenomena via relaxation and diffusion data, as we will show elsewhere. The data can be efficiently combined with spectral resolution by frequency-selective pulses (shown above), Hadamard-encoding^37^ or echo planar spectroscopic imaging type detection^38^. Moreover, we envision exploiting long-lived singlet-states^39^ in the ultrafast LNMR experiments in the investigation of slow diffusion and chemical exchange phenomena. The methods offer unprecedented opportunities to study fast processes in real-time, and to use various nuclear spin hyperpolarization techniques^9^,^10^,^11^ to increase the experimental sensitivity by several orders of magnitude. As demonstrated in this paper, the methods provide information about chemical structure and physical environments of molecules that is not available in the conventional NMR spectra, offering extremely interesting prospects, for example, for investigations of polymerization, gel formation, phases of ionic liquids, phase transitions, transport of substances into cells, metabolism and protein folding.

## Additional information

**How to cite this article:** Ahola, S. *et al.* Ultrafast multidimensional Laplace NMR for a rapid and sensitive chemical analysis. *Nat. Commun.* 6:8363 doi: 10.1038/ncomms9363 (2015).

## Supplementary Material

Supplementary Information Supplementary Figures 1-5, Supplementary Methods, Supplementary Discussion and Supplementary Reference
