# Supplementary material for: Ultrafast multidimensional Laplace NMR for a rapid and sensitive chemical analysis
Source: Nat Commun. 2015 Sep 18;6:8363. doi: 10.1038/ncomms9363 (PMC4595760; doi:10.1038/ncomms9363)
Supplement: Supplementary Information — Supplementary Figures 1-5, Supplementary Methods, Supplementary Discussion and Supplementary Reference [file ncomms9363-s1.pdf]

Supplementary Figures

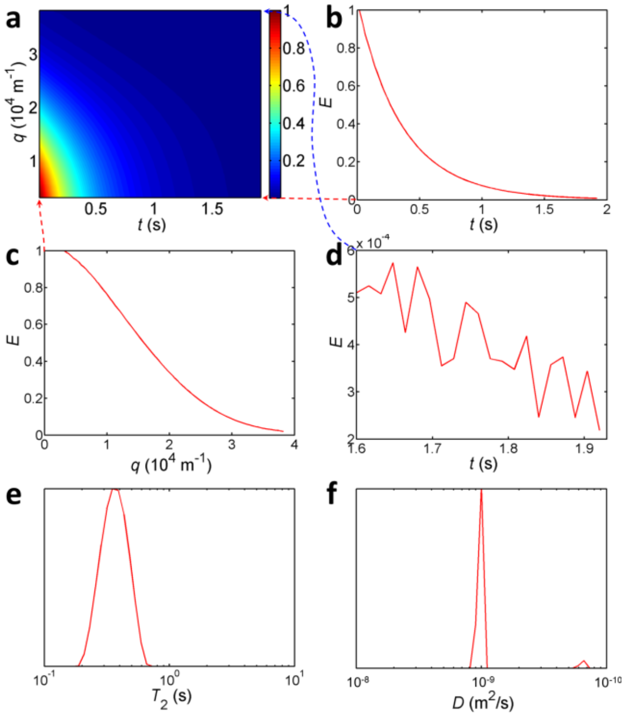

Supplementary Figure 1 | Ultrafast *D*-T_2 correlation experiment for the mixture of hexane and pentadecane. (**a**) Experimental data after the Fourier transform in the spatial frequency (k) dimension, the compensation of the spatial variations of the measurement coil sensitivity and the removal of the data not affected by the frequency sweep pulse. (**b**) The first row along the t direction. (**c**) The first column along the q direction ($q = \gamma\delta g/2\pi$, where γ is the gyromagnetic ratio, δ is the effective length of the diffusion gradient and g is the amplitude of the diffusion gradient). (**d**) The end portion of the last row, illustrating the noise level. SNR was estimated to be 8000. (**e**) 1D Laplace inversion of the first row. The resulting T_2 distribution includes only a single peak. (**f**) 1D Laplace inversion of the first column. The resulting D distribution includes only a single main peak (and an artificial peak at long D).

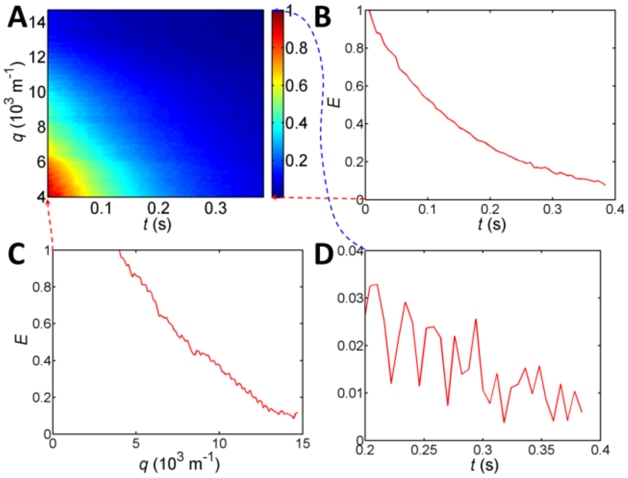

Supplementary Fig. 2 | Ultrafast *D*-T_2 correlation experiment of hyperpolarized propene. (**a**) Experimental data after the Fourier transform in the spatial frequency (*k*) dimension, the compensation of the spatial variations of the measurement coil sensitivity and the removal of the data not affected by the frequency sweep pulse. (**b**) The first row along the *t* direction. (**c**) The first column along the *q* direction. (**d**) The end portion of the last row, illustrating the noise level. SNR was estimated to be 80.

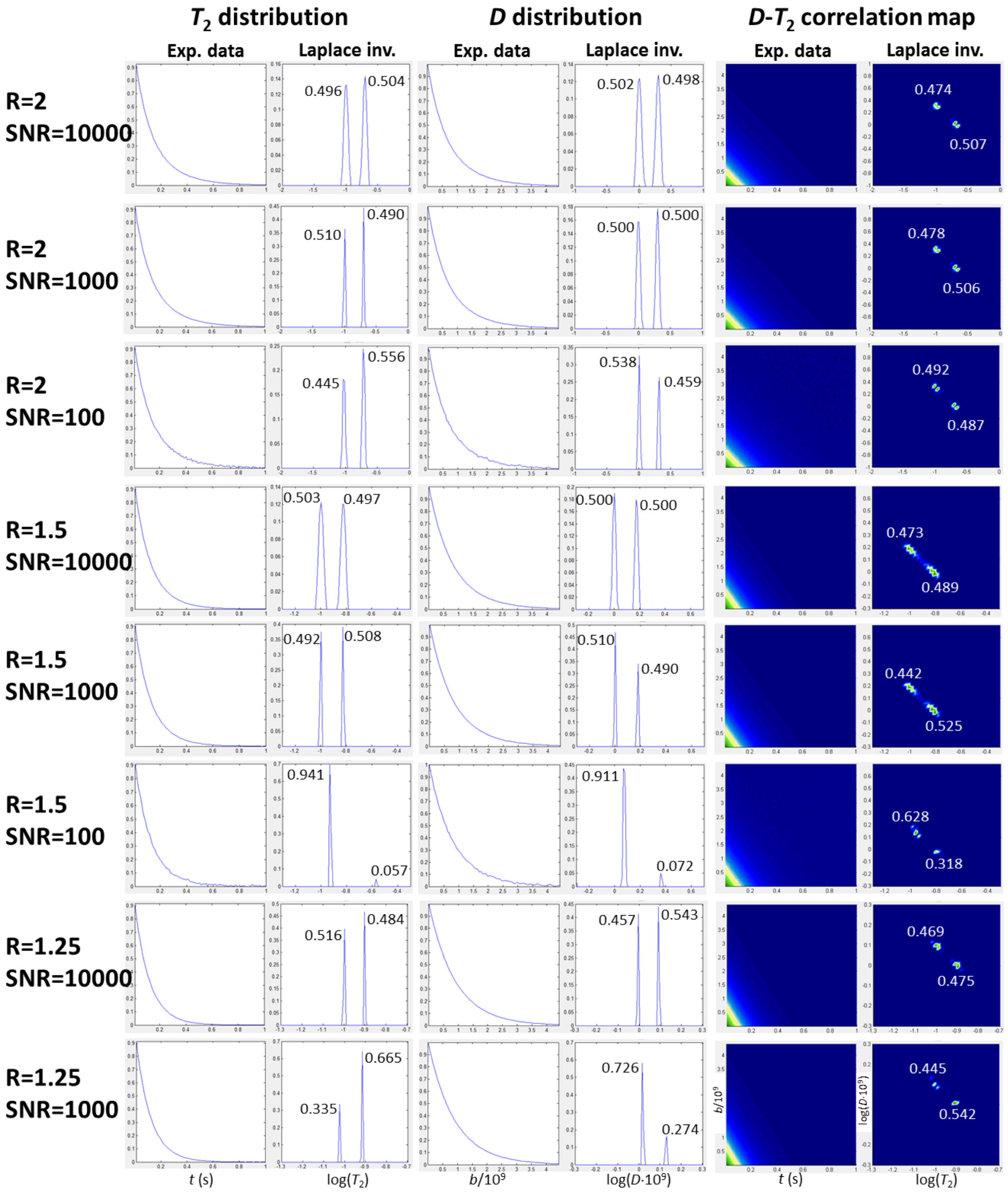

Supplementary Figure 3 | Simulated D-T_2 correlation LNMR data as well as 1D and 2D Laplace inversions for two components with equal (0.5 and 0.5) amplitudes. The T_2 relaxation time of component 1 is T_2^1 = 100 ms and diffusion coefficient of component 2 is $D_2 = 1 \cdot 10^{-9}$ m^2/s. The relaxation time and diffusion coefficient ratio $R = T_2^2/T_2^1 = D_1/D_2$ vary from 2 (top) to 1.25 (bottom), and signal-to-noise ratio (SNR) from 10000 to 100. The integrals of the signals are indicated in the distributions. Parameter $b = (\gamma G \delta)^2 \Delta$, where γ is the gyromagnetic ratio, G is the strength of the magnetic field gradient, δ is the length of the gradient pulse and Δ is the diffusion delay.

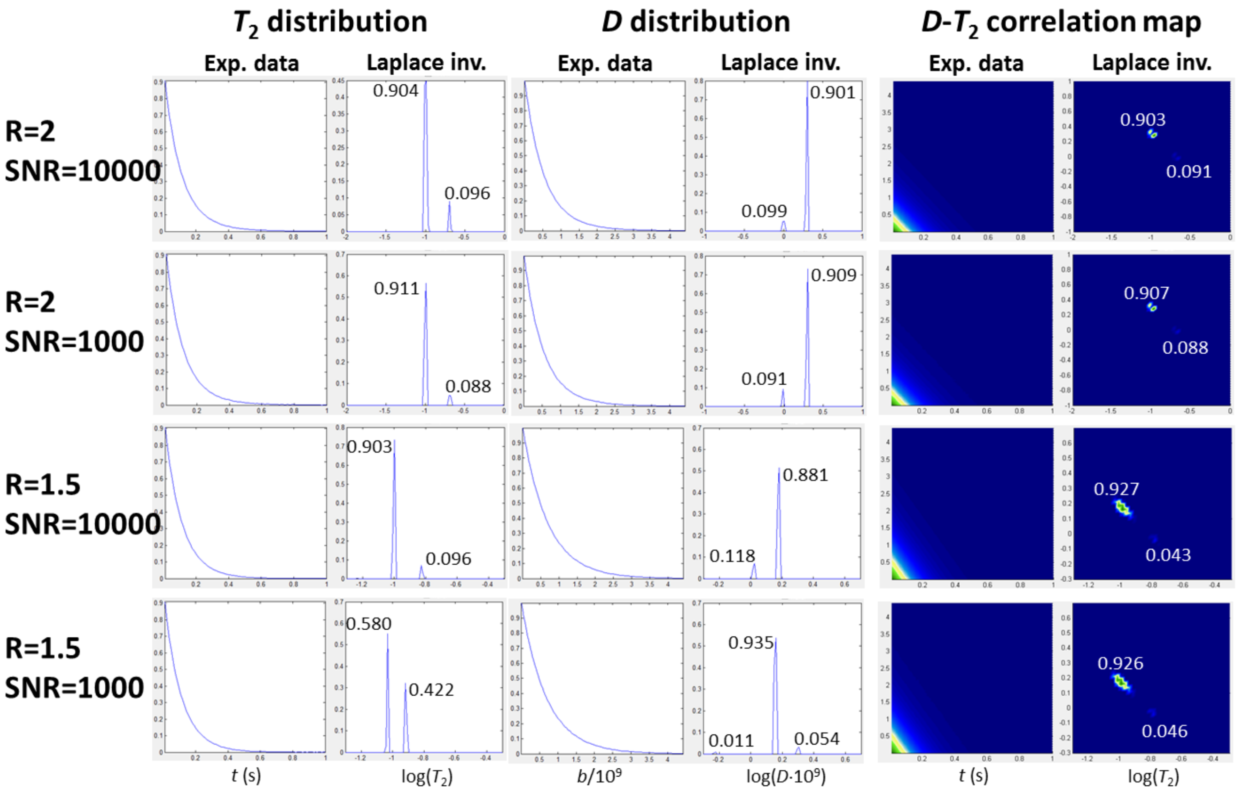

Supplementary Figure 4 | Simulated *D*-T_2 correlation LNMR data as well as 1D and 2D Laplace inversions for two components with 9:1 amplitude ratio (amplitudes are 0.9 and 0.1). The T_2 relaxation time of component 1 is $T_2^1 = 100$ ms and diffusion coefficient of component 2 is $D_2 = 1{\cdot}10^{-9}$ m^2/s. The relaxation time and diffusion coefficient ratio $R = T_2^2/T_2^1 = D_1/D_2$ vary from 2 (top) to 1.5 (bottom), and signal-to-noise ratio (SNR) from 10000 to 1000. The integrals of the signals are indicated in the distributions.

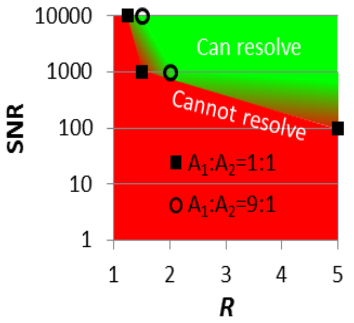

Supplementary Figure 5 | Illustration of LNMR SNR value required for reliable resolution of two components with narrow peaks, amplitude ratio of either 1:1 or 9:1 and relaxation time and/or diffusion coefficient ratio of *R*. The values are based on the results of the simulations shown in Figs. S3 and S4.

Supplementary Methods

Water in silica gel 60

Silica gel 60 (the average pore diameter of 6 nm and particle size of 60-200 μm, purchased from Merck, Darmstadt, Germany) was immersed in an excess of water (1% H_2O in D_2O) in a 10 mm sample tube.

The experiments were carried out on a Bruker Avance III 300 MHz spectrometer equipped with a micro-imaging unit at room temperature.

Parameters of the *ultrafast D-T_2 correlation experiment*: Relaxation delay 4 s, chirp (frequency-swept) pulse duration 1 ms, chirp pulse bandwidth 151 kHz, amplitude of gradient G_{sweep} = 27.9 G/cm, diffusion delay Δ = 50 ms, number of CPMG echoes 128, echo time 5 ms (t varying from 5 to 640 ms), amplitude of gradient G_{read} = 7.86 G/cm, field of view in the z direction 3 cm, number of points collected in each echo 512, number of accumulated scans 32, experiment time 2 min 31 s.

Parameters of the *conventional (reference) D-T_2 correlation experiment*: Relaxation delay 4 s, number of diffusion gradient steps 64, duration of diffusion gradient 2 ms, maximum diffusion gradient amplitude 49.13 G/cm, diffusion delay Δ = 50 ms, number of CPMG echoes 128, echo time 5 ms (t varying from 5 to 640 ms), number of accumulated scans 8, experiment time 41 min.

Hexane and pentadecane samples

Hexane sample composition: 167 μL hexane and 600 μL CCl_4 in a 5mm sample tube.

Pentadecane sample composition: 167 μL pentadecane and 600 μL CCl_4 in a 5mm sample tube.

Mixture composition: 167 μL hexane, 167 μL pentadecane and 600 μL CCl_4 in a 5mm sample tube.

The experiments were carried out on a Bruker Avance III 600 MHz spectrometer with a 5 mm BBO probe (equipped with a z gradient) at room temperature.

Parameters for the *ultrafast D-T_2 correlation experiment*: Relaxation delay 50 s, chirp (frequency-swept) pulse duration 1 ms, chirp pulse bandwidth 256 kHz, amplitude of gradient G_{sweep} = 88.43 G/cm, diffusion delay Δ = 70 ms, number of PROJECT echoes 120, echo time 16 ms (t varying from 16 to 1920 ms), amplitude of gradient G_{read} = 19.57 G/cm, field of view in the z direction 3 cm, number of points collected in each echo 512, number of accumulated scans 8 for the hexane and pentadecane samples and 128 for the mixture, experiment time 7 min for the hexane and pentadecane samples and 57 min for the mixture.

The data of the ultrafast D-T_2 correlation experiment as well as 1D T_2 and D distributions of the mixture are shown in Supplementary Fig. 1.

PHIP hyperpolarized propene

Parahydrogen-enriched H_2 (called simply parahydrogen) was produced by passing high purity hydrogen through 5 g of FeO(OH) ortho-para conversion catalyst kept at liquid nitrogen

temperature (77 K), which decreased the ortho-para spin isomer ratio from the room temperature equilibrium value of 3 : 1 to the value of 1 : 1. Propyne and parahydrogen were premixed in the 1 : 4 ratio in a 1 L gas cylinder with the total absolute gas pressure of 5 bar[1]. Before acquiring the data, the gas mixture was bubbled through a solution of $[Rh(COD)(DPPB)]BF_4$ catalyst in deuterated acetone in a 5 mm sample tube inside the NMR spectrometer.

The experiments were carried out on a Bruker Avance III 600 MHz spectrometer with a 5 mm BBO probe (including a z gradient) at room temperature.

Parameters of the *ultrafast D-T_2 correlation experiment with PHIP*: Relaxation delay 0 s, length of the selective (at 4.9 ppm) $\pi/2$ excitation pulse 10 ms, antiphase to inphase conversion delay $\tau_{anti\text{->}in}$ 25 ms, chirp (frequency-swept) pulse duration 1 ms, chirp pulse bandwidth 150 kHz, amplitude of gradient G_{sweep} = 20.05 G/cm, diffusion delay Δ = 70 ms, number of PROJECT echoes 64, echo time 6 ms (*t* varying from 6 to 384 ms), amplitude of gradient G_{read} = 19.57 G/cm, field of view in the z direction 3 cm, number of points collected in each echo 512, number of accumulated scans 1, experiment time 0.5 s.

The data of the ultrafast *D*-T_2 correlation experiment is shown in Supplementary Fig. 2.

DNP hyperpolarized DMSO

Parameters of the *ultrafast D-T_2 correlation experiment with ^1H DNP*: Stabilization time after injection of sample into flow cell 3 s, relaxation delay 0 s, chirp (frequency-swept) pulse duration 1 ms, chirp pulse bandwidth 107 kHz, amplitude of gradient G_{sweep} = 19.90 G/cm, diffusion delay Δ = 100 ms, number of CPMG echoes 64, echo time 10 ms (*t* varying from 10 to 640 ms), amplitude of gradient G_{read} = 7.83 G/cm, field of view in the z direction 3 cm, number of points collected in each echo 512, number of accumulated scans 1, experiment time 0.8 s.

Experimental method for the ultrafast D-T_2 correlation experiment with ^{13}C DNP: A 60 μL DMSO in D_2O (v/v 18:7) with 15 mM sodium salt of tris-8-carboxyl-2,2,6,6 tetrakis[2-(1-hydroxethyl)]-benzo(1,2-d:4,5-dS)bis(1,3)dithiole-4-ylmethyl free radical (OXO63; Oxford Instruments, Abingdon, U.K.) and 1 mM diethylenetriamine pentaacetic acid gadolinium complex (Gd-DTPA; Sigma-Aldrich, St. Louis, MO) was first hyperpolarized by irradiating 60 mW of microwaves at a frequency of 93.974 GHz at a temperature of 1.4 K in a field of 3.35 T for 3 hours. Subsequently, the sample was dissolved with superheated water. This sample solution was rapidly transferred to an injection loop, and driven into a flow cell in a 400 MHz NMR magnet using water from a high pressure pump.

Parameters of the ultrafast D-T_2 correlation experiment with ^{13}C DNP: Stabilization time after injection of sample into flow cell 3 s, relaxation delay 0 s, chirp (frequency-swept) pulse duration 2 ms, chirp pulse bandwidth 44.6 kHz, amplitude of gradient G_{sweep} = 50 G/cm, diffusion delay Δ = 200 ms, number of CPMG echoes 64, echo time 30 ms, amplitude of gradient G_{read} = 9.725 G/cm, field of view in the z direction 10 cm, number of points collected in each echo 512, number of accumulated scans 1, experiment time 3 s.

Parameters for ^{1}H diffusion measurement of thermally polarized samples: A pulsed field gradient stimulated echo (PFG-STE) sequence[2] was used to measure diffusion coefficients. The sequence consists of elements, [$d_1 - \pi/2 - G_1 - \pi/2 - \tau_2 - \pi/2 - G_2 - Acq$]. Here, "$G_1$", "$G_2$" and "*Acq*" refer to diffusion encoding gradient, diffusion decoding gradient and data acquisition. The relaxation delay is denoted as d_1. The sequence was repeated 16 times with amplitudes of both gradients simultaneously and linearly increased from 2% to 95% of the maximum strength; however, the duration of gradients were kept as 0.8 ms. The diffusion time (time between the first and last $\pi/2$ pulses) was set to 100 ms. The maximum z-gradient was separately calibrated by applying a PFG spin echo sequence[2] to a phantom.

Laplace inversion

Laplace inversion program provided by P. Callaghan (Victoria University of Wellington, New Zealand) was used for determining the 1D and 2D relaxation time and diffusion coefficient distributions[22]. The program is based on the method published by Venkataramanan *et al.*[6,7].

Supplementary Discussion

We simulated D-T_2 correlation LNMR data of two components with zero peak width, varying the peak amplitudes and separation as well as signal-to-noise ratio (SNR), and performed 1D and 2D Laplace inversions of the data. Supplementary Fig. 3 shows the results for two components with equal amplitudes and Supplementary Fig. 4 for two peaks with the amplitude ratio of 9:1.

Conclusions about the simulations with 1:1 amplitude ratio:

- If SNR is over 1000, peaks with relaxation time and diffusion coefficient ratio of $R = 1.5$ can be reliably resolved.
- If SNR is over 10000, peaks with $R = 1.25$ can be reliably resolved.

Conclusions about the simulations with 9:1 amplitude ratio:

- If SNR is over 1000, peaks with $R = 2$ can be reliably resolved.
- If SNR is over 10000, peaks with $R = 1.5$ can be reliably resolved.

These conclusions are visualized in Supplementary Fig. 5.

Details of creating 2D echo amplitude data for D-T_2 correlation simulations:

- Echo amplitude values of each of the two components were numerically calculated in Matlab by the following equation: $E_{\mathrm{i}}(k,l) = S_{max}C_i\exp[-4\pi^2 q(k)^2 D_i\Delta]\exp[-t(l)/T_{2i})$, where S_{max} is the total maximum amplitude of the signal, is C_i the portion of the component i (i = 1,2), q is the wave vector amplitude, D_i is the diffusion coefficient of component i, Δ is the diffusion delay, t is time and T_{2i} is the T_2 value of component i.

- The echo amplitude values of each of the two components were added together to get the total echo amplitude values $E(k,l)$
- 2D noise matrix was created by the MATLAB function “random”, returning random numbers with normal distribution
- The noise matrix was added to the 2D echo amplitude matrix
- Signal-to-noise ratio (SNR) was defined to be SNR = $S_{max}/(2\sigma_n)$, where σ_n is the standard deviation of noise

Supplementary Reference

1. Telkki, V.-V., Zhivonitko, V. V., Ahola, S., Kovtunov, K. V., Jokisaari, J., Koptyug, I. V. Microfluidic gas flow imaging utilizing parahydrogen-induced polarization and remote detection NMR. *Angew. Chem. Int. Ed.* **49**, 8363-8366 (2010).
